# Effect of the deuterium on efficiency and type of adipogenic differentiation of human adipose-derived stem cells *in vitro*

**DOI:** 10.1038/s41598-020-61983-3

**Published:** 2020-03-23

**Authors:** Alona V. Zlatska, Roman G. Vasyliev, Inna M. Gordiienko, Anzhela E. Rodnichenko, Maria A. Morozova, Maria A. Vulf, Dmytro O. Zubov, Svitlana N. Novikova, Larisa S. Litvinova, Tatiana V. Grebennikova, Igor A. Zlatskiy, Anton V. Syroeshkin

**Affiliations:** 1 State Institute of Genetic and Regenerative Medicine NAMS of Ukraine, 67 Vyshgorodska Str., Kyiv, 04114 Ukraine; 2 Biotechnology Laboratory ilaya.regeneration, Medical Company ilaya, 9 I. Kramskogo Str., Kyiv, 03115 Ukraine; 3 R.E. Kavetsky Institute of Experimental Pathology, Oncology and Radiobiology NAS of Ukraine, 45 Vasylkivska Str., Kyiv, 03022 Ukraine; 4 Peoples Friendship University of Russia (RUDN University), 6 Miklukho-Maklaya St, Moscow, 117198 Russian Federation; 5 Immanuel Kant Baltic federal University (IKBFU), 6 Gaidara St, Kaliningrad, 236001 Russian Federation; 6 Federal Research Center of Epidemiology and Microbiology named Gamalei, Moscow, Russian Federation

**Keywords:** Stem-cell biotechnology, Biotechnology, Stem cells

## Abstract

In this study, we performed an adipogenic differentiation of human adipose-derived stem cells (ADSCs) *in vitro* with different deuterium content (natural, low and high) in the culture medium during differentiation process with parallel analysis of the gene expression, metabolic activity and cell viability/toxicity. After ADSCs differentiation into adipocytes we have done the analysis of differentiation process efficiency and determined a type of resulting adipocytes (by morphology, gene expression, *UCP1* protein detection and adipokine production analysis). We have found that high (5 × 10^5^ ppm) deuterium content significantly inhibit *in vitro* adipogenic differentiation of human ADSCs compared to the groups with natural (150 ppm) and low (30 ppm) deuterium content. Importantly, protocol of differentiation used in our study leads to white adipocytes development in groups with natural (control) and high deuterium content, whereas deuterium-depleted differentiation medium leads to brown-like (beige) adipocytes formation. We have also remarked the direct impact of deuterium on the cellular survival and metabolic activity. Interesting, in deuterium depleted-medium, the cells had normal survival rate and high metabolic activity, whereas the inhibitory effect of deuterated medium on ADSCs differentiation at least was partly associated with deuterium cytotoxicity and inhibitory effect on metabolic activity. The inhibitory effect of deuterium on metabolic activity and the subsequent decrease in the effectiveness of adipogenic differentiation is probably associated with mitochondrial dysfunction. Thus, deuterium could be considered as an element that affects the substance chirality. These findings may be the basis for the development of new approaches in the treatment of obesity, metabolic syndrome and diabetes through the regulation of adipose-derived stem cell differentiation and adipocyte functions.

## Introduction

In the 21st century, non-communicable diseases (NCD) like obesity, metabolic syndrome and type 2 diabetes mellitus (T2DM) became the main medical problems of the humanity^1–4^. These diseases started in the Western world, but in parallel with the improving of human life standards, technological progress and the spread of the Western lifestyle also around the world. So, these diseases have become a global epidemic^5^. Currently, although there remains a correlation between the level of economic development and the frequency of these diseases, they have ceased to be a medical problem in high-income countries, but also have become an urgent item for the low-income and middle-income countries^6^.

A characteristic feature of obesity, metabolic syndrome and T2DM is the defection of glucose and lipids metabolism, which is manifested in insulin resistance, impaired fasting glucose, dyslipidemia, high blood sugar, high serum triglycerides, imbalance of different types of lipoproteins in blood serum^7,8^.

Prevention and treatment of these diseases include changing and controlling lifestyle, diet and the use of pharmaceuticals^9^. Despite the progress in medical science, pharmacology (the development of new substances for the correction of metabolism) and biotechnology (improving the process of insulin production), the solution to the problem of obesity, metabolic syndrome and T2DM requires new effective approaches.

It should be taken into account that adipose tissue is one of the key players in the development of obesity, metabolic syndrome and T2DM^10^. Conversely, adipose tissue can also be considered as the main target for the prevention and treatment of these pathological conditions^11^. The adipose tissue in the human body can be classified by anatomical location (subcutaneous, visceral, intermuscular, yellow bone marrow and breast), as well as functions (white and brown fat)^12^. The main function of white adipose tissue is to preserve energy in the form of lipids, insulating organs, and endocrine function - the production of hormones, growth factors, cytokines, chemokines and other biologically active substances that regulate energy metabolism and many other body functions, which were called adipokines^13^. The function of brown adipose tissue is heat production during adaptive thermogenesis. In humans, unlike rodents (laboratory animals most widely used in medical experiments, including modeling of obesity, metabolic syndrome and T2DM), brown adipose tissue is present in significant amount only in newborns and infants^14^. Recently, the existence of active thermogenic adipose tissue in human adults was shown, but this adipose tissue differs from the classical brown adipose tissue in a number of aspects (development, morphology, gene expression, adipokine production etc)^15^. This adipose tissue is called “beige” or “brite” (brown in white) adipose tissue. All types of adipocytes arise from adipose-derived stem cells (ADSCs) in the process of differentiation. At present, a number of questions regarding the origin of beige adipocytes (from the same stem cell as white adipocytes, or from the same stem cell as brown adipocytes, or from own stem cell) remain debatable as well as the ability of white adipose tissue to transdifferentiate into brown/beige adipose tissue^16^.

The ability to control the formation of new adipose tissue, to convert white adipose tissue into brown/beige adipose tissue, or to set the direction of differentiation of ADSCs into a specific subtype of adipocytes is an attractive target for the development of new pharmacological substances to treat obesity, metabolic syndrome and T2DM^17^.

In addition to the search for new pharmacological substances targeted to adipose tissue functions and/or various other biochemical aspects of energy homeostasis, it is also important to study the role of water in human health, metabolism, and pathogenesis of different diseases. Water is the most common chemical substance on the Earth and constitute the largest mass part of living organisms in percentage ratio. Water is also a universal solvent in which the basic biochemical processes of living organisms occur. An important component of a healthy diet is consumption of drinking water instead of sugar-containing and carbonated beverages^18,19^. So, the modulation of biological and physicochemical properties of water is also a promising opportunity to improve the effectiveness of treatment for obesity, metabolic syndrome and T2DM.

Deuterated water has the same chemical formula as ordinary water, but instead of two atoms of a light hydrogen isotope (protium) it contains two atoms of a heavy hydrogen isotope (deuterium). Deuterated water shows only slight cytotoxicity. In general, chemical reactions in deuterated water have slower rate than in ordinary water^20–23^.

Deuterium can be considered as a regulator of the biological properties of normal and/or cancer cells^24–27^. One of the medicine trends is the development of deuterium-containing drugs^28–30^. The other direction refers to the role of isotopology D/H ratio and its change in water to be used as an adjuvant in cancer treatment^31–34^. Different D/H ratio is manifested in the form of kinetic isotope effect, which is characterized by a change in the biotransformation and excretion rates of the drugs^35–37^. Moreover, the methodological approaches for drugs quality control based on water isotopology, could reduce the toxic load of the body^38,39^.

In our previous studies, we have shown that different deuterium concentration in growth medium affects the proliferation, migration and metabolic activity of cultured human adipose-derived stem cells (ADSCs)^40^.

In present study, we consider the question whether deuterium is involved in regulation of ADSCs differentiation, different types of adipocytes development and an adipokine production by adipocytes. Adipogenic differentiation of ADSCs was chosen as a model for our *in vitro* pilot study, where we compared the efficiency of adipogenesis in media with different deuterium contents (natural, low and high).

## Materials and Methods

### Types of water used in the study. Water preparing and testing (characterization)

The following basic water samples with various deuterium content were used in this study: deuterium-depleted water (ddw) with D/H = 4 ± 2 ppm D (Sigma-Aldrich, USA); deuterated water (D_2_O) with D/H = 99 absolute at. % D (Sigma-Aldrich, USA). Growth media with various deuterium content were prepared by diluting deuterium-depleted and deuterated water. The following media for adipogenic differentiation were used in this study: № 1 - medium with D/H ratio 30 ppm; № 2 - the medium with the highest deuterium content - D/H ratio 500.000 ppm. The milliQ water (MilliQ-system, UK) served as a standard (control) with D/H ratio 150 ppm. MilliQ, deuterium-depleted and deuterated water had no differences in physical characteristics or in trace element composition, except the deuterium content. This excluded the multifactor influence in the system for all comparison groups. Detailed description of the method was presented in previous studies^22,27,40,41^.

The deuterium content was controlled by multipass laser absorption spectroscopy on the Isotopic Water Analyzer-912-0032 (Los Gatos Research Inc., USA). Detailed description of this method was presented in previous studies^22,27^.

Chemical analysis of water with various deuterium content was performed by inductively coupled plasma-mass spectrometry on the ICP-QMS Agilent 7500CE spectrometer (Agilent Technologies, USA). Detailed description of the method was presented in previous studies^22,27^. Calibration solutions with a high range of elements’ concentration (from 0.1 μg/L to 100 μg/L) were used for the device calibration. The solutions were prepared with the international standard 2.74473.0100 “ICP Multi Element Standard Solution XXI CertiPUR” which contains the following elements: Ag, Al, As, Ba, Be, Bi, Ca, Cd, Co, Cr, Cs, Cu, Fe, Ga, In, K, Li, Mg, Mn, Na, Ni, Pb, Rb, Se, Sr, Tl, U, V, Zn, Hg. The concentration of all above-listed 24 elements in the milliQ, deuterium-depleted or heavy water did not exceed the upper detection limit (detection limit range – 0.1–10 ppm).

### The ADSCs cultivation (*in vitro* live cell experiment)

The experiments with use of human cell culture *in vitro* were carried out in accordance with the human experiment issues of the Code of Ethics of the World Medical Association (Declaration of Helsinki). All procedures related to obtaining human biopsies, cell isolation and culturing were performed with written informed donor consent and in accordance with the laws of Ukraine. The study protocol was approved by the Bioethics Committee of the State Institute of Genetic and Regenerative Medicine NAMS of Ukraine (Kyiv, Ukraine). The cell culturing was carried out in the GMP/GTP-compliant biotechnological laboratory ilaya.regeneration (License to operate the Banks of human cord blood, other tissues and cells; issued by the Ministry of Health of Ukraine AE No. 186342 from 11.07.2018).

In all cases the voluntary informed consents were signed by donors of ADSCs. ADSCs samples (n = 6) were obtained from abdominal subcutaneous fat tissue of the healthy donors (3 female and 3 male) with normal somatometric and biochemical parameters without signs of obesity and viral or microbial infection. The age of patients was 23 ± 4.0 years. Body mass index (BMI) of adipose tissue donors was 20 ± 1.3.

The ADSCs were isolated from the lipoaspirate by enzymatic digestion in 0.1% collagenase IA and 0.1% pronase with 2% fetal bovine serum (FBS) (all - Sigma-Aldrich, USA) for 1 h at 37 °С. Detailed description of the method was presented in previous studies^40–46^. The obtained cell suspension was transferred to 25 cm^2^ cell culture flask (SPL, Korea) and cultured in the following growth medium: modified МЕМ-α (Sigma-Aldrich, USA) prepared from the powder diluted with milliQ water of natural isotope content supplemented with 10% FBS (Sigma-Aldrich, USA), 2mM L-glutamine, 100 U/ml penicillin, 100 μg/ml streptomycin and 1 ng/ml bFGF-2 (all from Sigma-Aldrich, USA). The cells were cultured in multi-gas incubator CB210 (Binder, Germany) at +37 °C in the atmosphere of saturated humidity, 5% СО_2_ and 5% О_2_.

Before using in experement ADSCs were expanded until passage 3 and characterized according to criteria of International Society of Cellular Therapy^42–45,47^.

### Flow cytometry for the ADSCs phenotype determination

The cell phenotype was assessed by flow cytometry (FACS) on BD FACSAria fluorescence-activated flow cell sorter-cytometer (BD Pharmingen, BD Horizon USA) and was performed in accordance to monoclonal antibodies manufacturer’s instructions (BD Pharmingen, BD Horizon USA). BD FACS Diva 6.1 software (BD Pharmingen, BD Horizon USA) was used for analysis. Detailed description of the method was presented in previous studies^41,46^.

### Directed osteogenic differentiation of ADSCs

Osteogenic differentiation was performed in DMEM medium with low glucose (1 g/L) (BioWest, France) prepared from the powder by dissolution with milliQ water of natural isotope content and supplemented with addition of 10% FBS, 100 nM dexamethasone, 10 mM β-glycerophosphate, 500 μg/ml ascorbate-2-phosphate (all – Sigma-Aldrich, USA). After 21 days fixation and staining of cells with Alizarin Red S were carried out on mineral deposits. Detailed description of the method was presented in previous studies^46^.

### Directed chondrogenic differentiation of ADSCs

The evaluation of chondrogenic differentiation of ADSCs was carried out using the micromass culture method. For this, 500 000 cells were centrifuged for 10 min at 400 g in 15 ml test tubes (Nunc, USA). Further, the cell precipitate was cultured in a chondrogenic induction medium containing DMEM with 4.5 g/L glucose (BioWest, France) prepared from the powder by dissolution with milliQ water of natural isotope content supplemented with addition of 50 μg/ml ascorbate-2-phosphate, 40 μg/L L-proline, 100 μg/ml pyruvate sodium, 10 ng/ml rhTGF-β3, 10–7 M dexamethasone (all – Sigma-Aldrich, USA), 1% ITS supplement (all – Gibco, USA), 1.25 mg/ml of bovine serum albumin, BSA (BioWest, France). Detailed description of the method was presented in previous studies^46^.

### Directed adipogenic differentiation of ADSCs

The ADSCs were seeded with a density of 30 000 cells per 1 cm^2^. In 24 h the growth medium was changed on the serum-free adipogenic differentiation media with low, natural and high deuterium content: (1) phase I induction (4 days) – DMEM high glucose (4.5 g/L) (BioWest, France) prepared from the powder by dissolution with milliQ water of natural isotope content and supplemented with: 1 µM dexamethasone, 0.1 µM hydrocortisone, 50 µM indomethacin, 250 µM isobutylmethylxanthine, 0.2 nM triiodothyronine, 5 µg/ml insulin, 1% fatty-acid mixture, 0.01% bovine serum albumin (BSA), 1% ITS supplement, 50 µM ascorbate-2 phosphate, 2 mM L-glutamine, (all – Sigma-Aldrich, USA); phase II differentiation (10 days): media with same content without dexamethasone, indomethacin and isobutylmethylxanthine. Control adipogenic induction and differentiation media had a natural deuterium content. Experimental growth media had a composition similar to the control one, but was prepared on deuterated and deuterium-depleted waters. Gene expression, viability and metabolic activity of the cells were analyzed on days 3, 7 and 14 after induction to assess ADSCs adipogenic differentiation.

### Cytochemistry, immunocytochemistry and histochemistry

Detailed description of the method was presented in previous studies^46^. Briefly, to confirm the osteogenic and adipogenic differentiation, the cells were fixed for 20 min in 10% buffered formalin (Sigma, USA), washed with DPBS (Sigma-Aldrich, USA) and stained for 20 min with 2% solution of Alizarin Red S (pH 4.1; for detecting calcified extracellular matrix deposits) or 0.5% solution of Oil Red O (for staining of neutral lipids) and Romanowsky-Giemsa stain for counterstaining, respectively (all – Sigma-Aldrich, USA).

The Oil Red O extraction was performed as described previously^48^. Measurements were performed on a LabSystems Multiskan PLUS spectrofluorometer (USA).

To confirm chondrogenic differentiation spheroids were removed from the incubator after 21 days of culturing, the medium was carefully aspirated, and spheroids were washed twice with DPBS. Spheroids were fixed in 10% buffered formalin for 2 hrs, dehydrated with ethanol, cleared with xylen and finally embedded in paraffin. Then, they were sectioned at 5 µm thickness. Slides were washed twice with distilled water and were stained by filtered Alcian Blue staining solution in the dark for 45 minutes at room temperature. Staining solution was removed and washed twice with PBS (all – Sigma-Aldrich, USA). Cartilage became stained an intense blue^46^.

The following primary antibodies were used for immunocytochemical staining: rabbit polyclonal against USP1 (Invitrogen, USA) and mouse monoclonal against PERILIPIN (R&D, USA). Secondary antibodies were donkey anti-mouse Alexa-488 and donkey anti-rabbit Alexa-647 conjugated (Thermo Fisher, USA). The cells were fixed for 20 minutes with cold 4% paraformaldehyde, permeabilized with intracellular staining for 15 min with 0.1% Triton X-100, blocked for 30 min in phosphate buffered saline with 0.1% Tween-20, 1% BSA, 5% FBS. The slides were incubated with primary antibodies overnight at 40 °C and with secondary antibodies for 1 hour at room temperature.

### Cell viability

For the viability/cytotoxicity assessment, the cells were stained with РI (propidium iodide) (Sigma-Aldrich, USA) and FDA (fluorescein diacetate) (Sigma-Aldrich, USA) before differentiation and after 3, 7 and 14 days of adipogenic differentiation. Detailed description of the method was presented in previous studies^40,41^. The number of dead and living ADSCs in different groups was counted using inverted fluorescent microscopy AxioObserver A1 and ZEN 2012 software (Carl Zeiss). The cell viability was calculated as the ratio of living cells to the total number of cells and was expressed as a percentage according to the formula:

$${\rm{Viability}}=({\rm{number}}\,{\rm{of}}\,{\rm{living}}\,{\rm{cells}}/{\rm{total}}\,{\rm{number}}\,{\rm{of}}\,{\rm{cells}})\times 100 \% $$

### The ADSCs metabolic activity

Detailed description of the method was presented in previous studies^41,49^. ADSCs metabolic activity was assessed on days 3, 7 and 14 of adipogenic differentiation. 10% of Alamar Blue (redox indicator; Thermo Fisher, USA) was added to the culture medium and incubated for 3 h according to manufacturer’s instructions. Reduced Alamar Blue was detected at 540 nm vs 630 nm at LabSystems Multiskan PLUS spectrofluorometer (USA). Cell metabolic activity was calculated according to the following formula:

$$ \% \,{\rm{of}}\,{\rm{reduction}}=(({\rm{\varepsilon }}{\rm{ox}})\,{\rm{\lambda }}2\cdot {\rm{A}}{\rm{\lambda }}1-({\rm{\varepsilon }}{\rm{ox}})\,{\rm{\lambda }}1\cdot {\rm{A}}{\rm{\lambda }}2){\rm{experiment}}/(({\rm{\varepsilon }}{\rm{ox}})\,{\rm{\lambda }}2\cdot {\rm{A}}{\prime} {\rm{\lambda }}1-({\rm{\varepsilon }}{\rm{ox}})\,\lambda 1\cdot {\rm{A}}{\prime} {\rm{\lambda }}2){\rm{control}};$$

where λ1 = 540 nm, λ2 = 630 nm

(εox) λ2 = 34,798

(εox) λ1 = 47,619

Aλ1 – experimental sample absorption at λ1 = 540 nm

Aλ2 – experimental sample absorption at λ2 = 630 nm

A′ λ1 – control sample absorption at λ1 = 540 nm

A′ λ2 - control sample absorption at λ1 = 630 nm

### The RT-qPCR assay

Detailed description of the method was presented in previous studies^46^. Total RNA was isolated from ADSCs using NucleoZOL (MACHEREY-NAGEL GmbH & Co. KG, Germany) according to manufacturer’s protocol. The RNA quality and concentration were determined with a spectrophotometer NanoDrop 1000 (Thermo Scientific, USA). 2 μg of isolated RNA were reverse transcribed to cDNA using RevertAid First Strand cDNA Synthesis kit (Thermo Scientific, USA). RT-qPCR was performed with a 7500 Real-Time PCR System (Applied Biosystems, CA, USA) using 5× HOT FIREPol EvaGreen qPCR Mix Plus (ROX) (Solis BioDyne, Estonia). The Applied Biosystems 7500 system software (V. 1.3.1) was used for data analysis. The primers sequences are listed in Table 1. The following PCR cycling conditions were applied: 10 min at 95 °C, 40 cycles of 10 s at 95 °C, and 40 min at 60 °C. Expression level of TATA-box binding protein (TBP) was used as internal control. Ct values for target genes were normalized against Ct value of TBP at the same threshold level. The relative quantification (comparative Ct (ΔΔCt) method) was used to compare the expression levels of the target genes with the internal control. Dissociation curve analysis was performed after every run to check the primers specificity. Results were presented in relative units. For all conditions, reaction was performed three times (each gene in triplicate). GraphPad Prism 4 (GraphPad Software, USA) and MS Excel were used for statistical analysis and graphic data presentation.

**Table 1:** The list of primer sequences used in this study.

№	Gene	Forward Primer (5′-3′)	Reverse Primer (5′-3′)	Product length
1	*TB*P	ccactcacagactctcacaac	ctgcggtacaatcccagaact	
2	*FABP4*	acaggaaagtcaagagcaccat	aactctcgtggaagtgacgc	152 bp
3	*PPARG*	agcctcatgaagagccttcca	tccggaagaaacccttgca	120 bp
4	*LEP*	agggagaccgagcgctttc	tgcatctccacacaccaaacc	122 bp
5	*ADIPONECTIN*	gcagtctgtggttctgattccatac	gcccttgagtcgtggtttcc	112 bp
6	*LPL*	tggaggtacttttcagccaggat	tcgtgggagcacttcactagc	102 bp
7	*UCP1*	gtgtgcccaactgtgcaatg	ccaggatccaagtcgcaaga	95 bp

### Adipokine production

After differentiation ADSCs in adipocytes, the content of the following 10 adipokines, cytokines and chemokines were determined in the incubation medium by the method of flow fluorimetry (multiplex analysis, Luminex xMAP): LEPTIN, ADIPONECTIN, ADIPSIN, VASPIN, CHEMERIN, TNF-α, IL-6, IL-8, IL-10, МСР-1, IP-10. This method included a multiplex immune reaction that took place on the various-diameter microparticles carrying the absorbed antibodies, and subsequent flow fluorescence analysis as well as simultaneous measurement of cytokine concentration. The procedures were conducted according to the Bio-Plex Pro Assay protocol. The results were recorded using an automatic photometer for Bio-Plex microplates (Bio-Plex 200 Systems, Bio-Rad, USA) and the Bio-Plex Manager (“Bio-Rad”) software. The test substances concentration was determined from the calibration curve for each cytokine (the dynamic range 2–32 000 pg/mL) according to the manufacturer recommendations. Detailed description of the methods was presented in previous studies^46^.

### Microscopy

Microscopy examinations of live cells, cell cultures and cytological slides were carried out with inverted AxioObserver A1 microscope equipped with an AxioCam ERc 5 s digital camera (all – Carl Zeiss, Germany) and ZEN 2012 software.

### Statistics

The data were reported as mean ± SD for each group. Statistical analyses were performed using one-way analysis of variance (ANOVA) in Origin Pro software. Differences were considered to be statistically significant when p < 0.05.

## Results

### Preparation and characterization of ADSCs

Subcutaneous fat is a convenient source for ADSCs obtaining^50^. The possibility of *in vivo* converting white adipocytes of subcutaneous adipose tissue into beige/brown adipocytes was previously shown^51^. Thus, the *in vitro* study of ADSCs differentiation from subcutaneous adipose tissue is a valuable tool in the search for new substances targeted on adipocyte function and in the development of new methods for obesity, metabolic syndrome and T2DM treatment.

Cells obtained from human lipoaspirate adherent were expanded *in vitro* until P3 and before experiments were characterized according to the ISCT position papers for compliance to minimal defined criteria of MSCs and ADSCs^47^.

**Figure 1:**
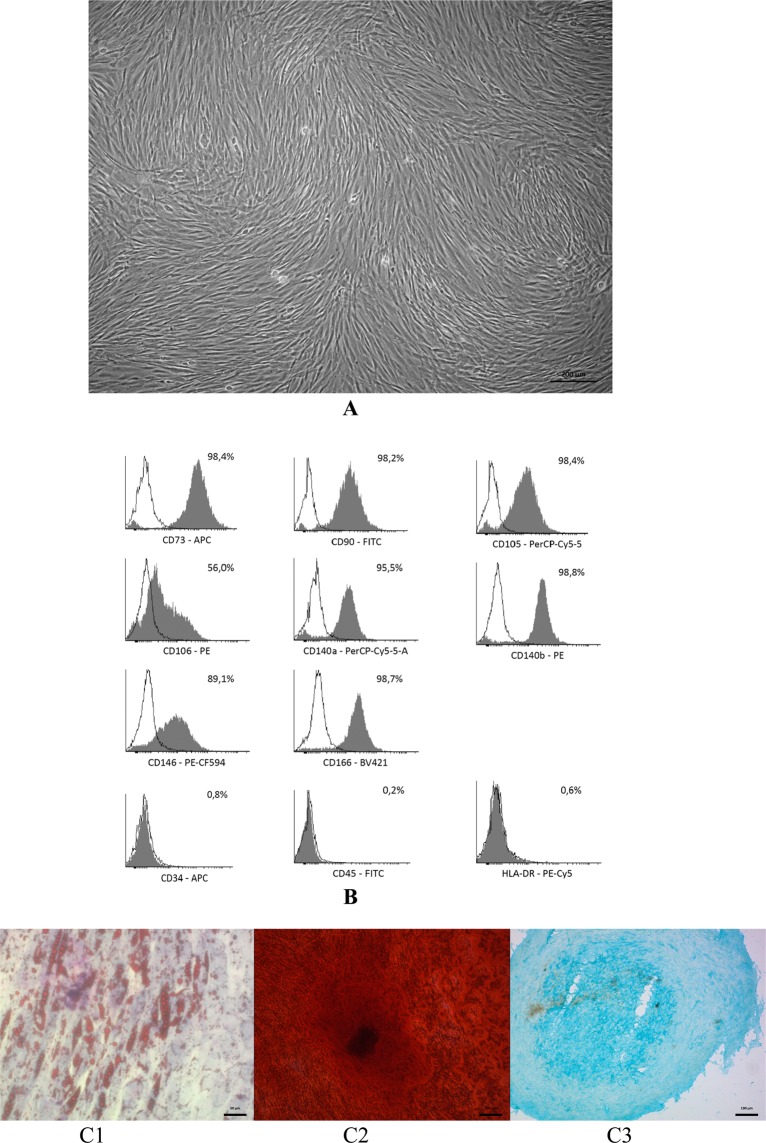
Сharacterization of ADSCs. (**A**) The ADSCs morphology at P3 before directed multilineage differentiation. Phase-contrast microscopy. Bar scale – 200 µm. (**B**) Representative FACS histograms the ADSCs immunophenotype at P3. (**C**) The ability to direct three-lineage differentiation of ADSCs *in vitro*. C1 – adipogenic differentiation, Oil Red and Romanovsky-Giemsa stain, the bar = 50 µm; C2 – osteogenic differentiation, Alizarin Red S stain, the bar = 100 µm; C3 – chondrogenic differentiation, Alcian Blue stain, bar scale = 100 µm.

After expansion over three passages, the cells obtained from lipoaspirate presented homogeneous population of cells with fibroblast-like morphology (Fig. 1A). The study of ADSCs immunophenotype showed that they expressed characteristic positive stem cell markers and did not express negative markers (Fig. 1B).

Also, ADSCs demonstrated key characteristic features of MSCs – the ability to directed orthodoxic three-lineage differentiation *in vitro*. So, after 14 days of cultivation in adipogenic differentiation medium, the fibroblast-like cells were converted into adipocytes containing lipid vacuoles (Fig. 1C1). The ADSCs also demonstrated ability to osteogenic differentiation and production of mineralized extracellular matrix. Alizarin Red S staining showed a positive reaction to the Ca content at day 21 after differentiation (Fig. 1C2). As for the chondrogenic differentiation, a dense chondroid was obtained after 21 days of chondrogenic induction (Fig. 1C3).

Thus, ADSCs isolated from lipoaspirate, complied the minimal ISCT criteria for MSCs, such as adherence to plastic in standard culture conditions, fibroblast-like morphology, typical phenotype and ability for directed orthodoxic multilineage differentiation *in vitro*.

To find out whether deuterium could be involved in regulation of adipogenesis, we composed adipogenic differentiation media on the base of deuterium-depleted and deuterated water. The adipogenic differentiation medium, that was made with milliQ water, served as control.

### Directed adipogenic differentiation of ADSCs with various deuterium content

Differentiation of ADSCs into adipocytes is a complex multi-step process in which the stages of induction/commitment (adipocyte progenitors, days 0–3), early differentiation (pre-adipocytes, days 3–7) and terminal differentiation (mature adipocytes, days 7–14) can be distinguished.

**Figure 2:**
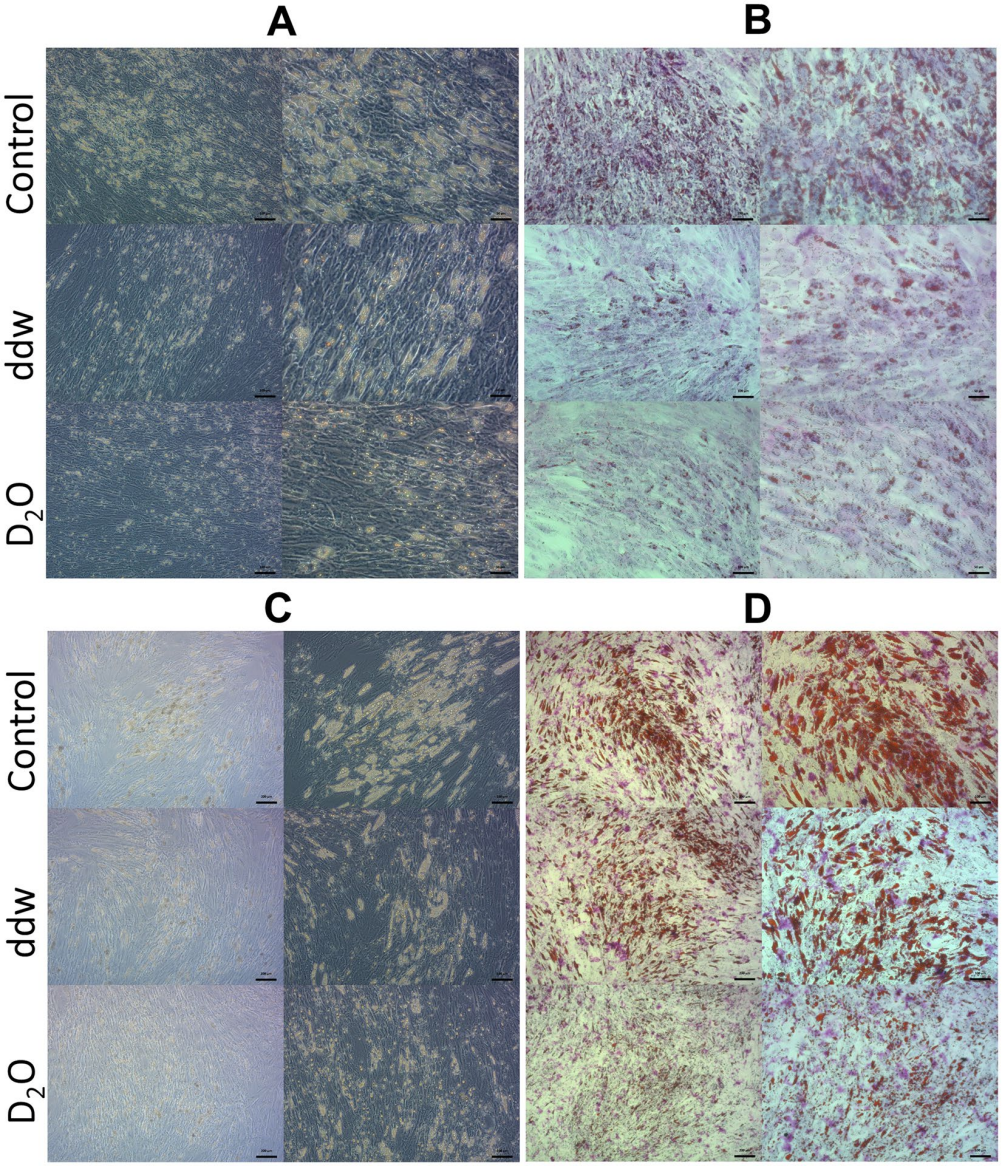
Adipogenic differentiation of ADSCs in media with various deuterium concentration. (**A**) Phase-contrast microscopy 7 days, (**B**) Light microscopy Oil Red O dye 7 days, (**C**) Phase-contrast microscopy 14 days, (**D**) light microscopy Oil Red O dye 14 days). Control – differentiation medium was made on the base of milliQ water; ddw – differentiation medium was made on the base of deuterium-depleted water; D_2_O – differentiation medium was made on the base of deuterated water. In each section: left column – the bar = 100 µm; right column – the bar = 50 µm.

After 7 days of cultivation in the adipogenic differentiation medium, cells began to accumulate lipid vacuoles in all groups (Fig. 2A,B). However, there were noticeable differences in number, size and form of the lipid vacuoles in ADSCs that underwent differentiation in deuterated and deuterium-depleted inductive media compare to control. A significant number of cells with lipid vacuoles were observed in the control group, and only single adipocytes in the experimental groups. At the same time, the process of differentiation had no morphological differences between groups and followed the path of adipocyte formation with the fusion of the initial small lipid vacuoles into one or two large vacuoles located centrally in the cytoplasm, which is typical for white adipocytes.

But on the 14^th^ day there were no such a striking difference except for the group of deuterated differentiation medium, where lipid vacuoles were much smaller compared to other groups (Fig. 2C,D). As for the cells in deuterium depleted medium, they almost reached the control ones.

At the same time, on the 14th day, significant morphological differences were observed between differentiated from ADSCs adipocytes in all groups. Thus, in the control group (natural deuterium content) and in the group with high deuterium content, predominantly unilocular adipocytes containing one or two large lipid vacuoles were formed. As indicated earlier, this morphological trait is a characteristic feature of white adipocytes. In the group with low deuterium content, single unilocular adipocytes were also found. However, most adipocytes had numerous small vacuoles located on the periphery of the cytoplasm. This morphology is characteristic for brown and beige adipocytes.

**Figure 3:**
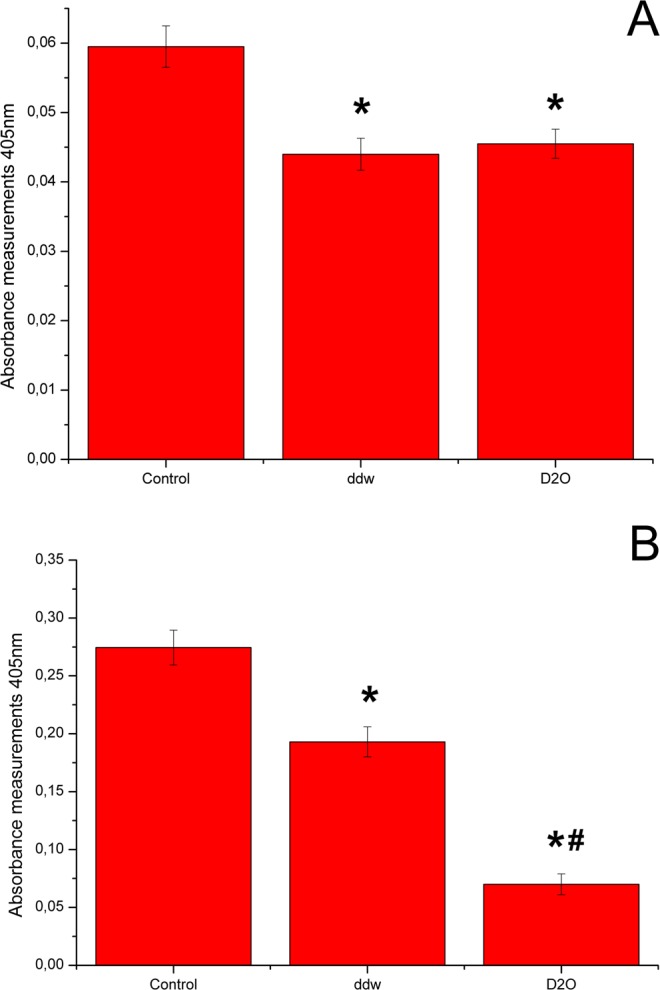
Diagrams represent results of Oil Red extraction after 7 (**A**) and 14 (**B**) days cultivation in adipo-inductive media with various deuterium content. Control – differentiation medium was made on the base of milliQ water; ddw – differentiation medium was made on the base of deuterium-depleted water; D_2_O – differentiation medium was made on the base of deuterated water. (The results are expressed as mean ± SD (n = 6), significant differences between groups: *p < 0.05 compared to control group, #p < 0.05 compared to ddw group).

The adipogenic differentiation efficiency was evaluated by Red Oil O dye extraction on the 7th аnd 14th days of the experiment (Fig. 3). Both high and low doses of deuterium inhibited adipogenic differentiation of ADSCs. On day 7, inhibition of adipogenic differentiation of ADSCs was the same in deuterium-depleted and deuterated media (Fig. 3A). However, adipogenic media on the base of deuterated water showed higher differentiation inhibition on day 14 (Fig. 3B).

### ADSCs gene expression profile in process of adipogenic differentiation in media with various deuterium content

**Figure 4:**
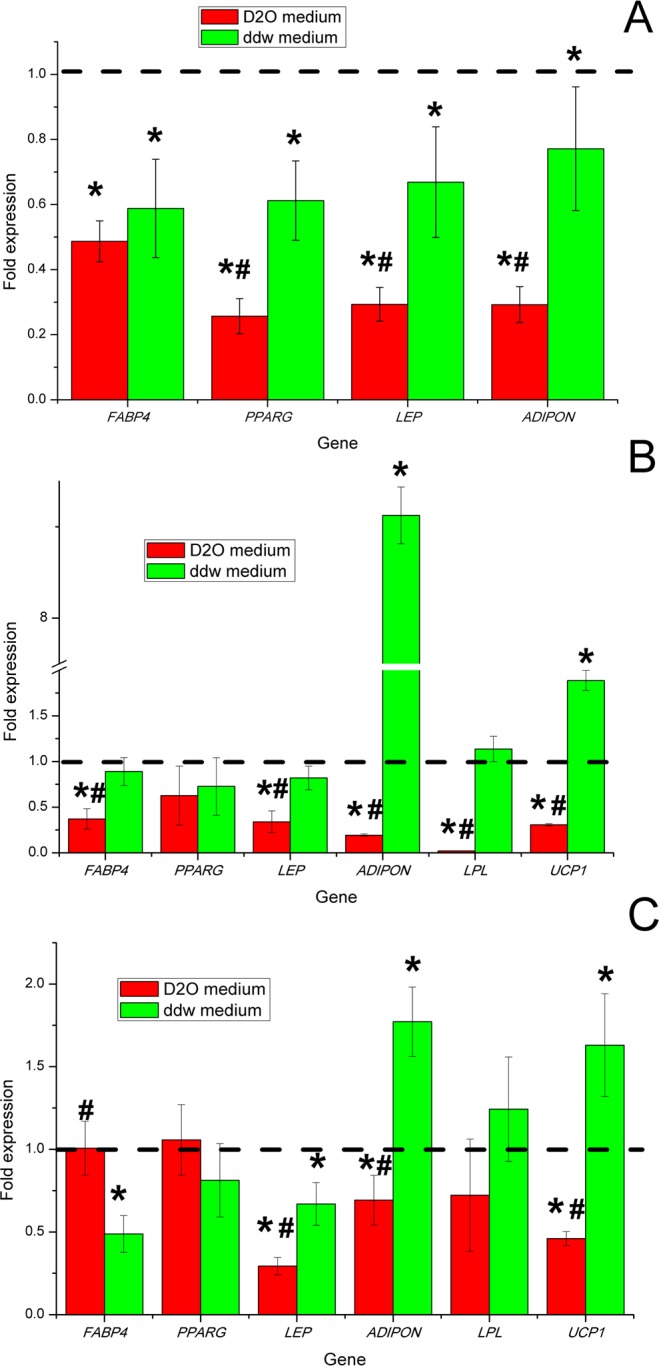
Diagrams represent results of the mRNA expression level of adipogenic markers in ADSCs after adipogenic differentiation in media with various deuterium content after 3 (**A**), 7 (**B**) and 14 (**C**) days. Control – differentiation medium was made on the base of milliQ water, marked with a dotted line; ddw – differentiation medium was made on the base of deuterium-depleted water; D_2_O – differentiation medium was made on the base of deuterated water. (The results are presented as mean ± SD (n = 6), significant differences between groups: *p < 0.05 compared to control group, #p < 0.05 compared to ddw group).

To obtain further insight into the molecular mechanisms of ADSCs during adipogenic differentiation in differentiation media with various deuterium content, we evaluated the mRNA expression levels of the key genes linked with adipogenesis. Evaluation of gene expression by cells was performed before the experiment and on days 3, 7 and 14. (Fig. 4). mRNA expression of all genes was normalized to TBP expression level. The fold-change was calculated over the control conditions that correspond to 1.

The ADSCs did not express the studied key adipogenesis genes in culture *in vitro* before differentiation (data not showed). At day 3 after starting differentiation ADSCs began to express master-gene of adipogenesis *PPARG* and other characteristics for adipocytes genes: *FABP4* (encodes carrier protein for fatty-acid), *LEP* and *ADIPON (*key adipokines that *in vivo* regulate satiety, hunger and various aspects of energy metabolism in human body). At this point, expression of *LPL* and *UCP1* genes was not detected, which coincided with morphological signs of the formation of immature white adipocytes. Thus, at the induction/commitment stage, the expression of adipogenesis genes was statistically significantly higher in the control group with natural deuterium content (Fig. 4A). According to the data on gene expression in groups with low and high deuterium content, inhibition of the induction/commit process of ADSCs in the adipogenic direction occurred.

Based on gene expression analysis, at day 7^th^ ADSCs adipogenic differentiation in deuterium-depleted medium was only partially inhibited comparing to control differentiation medium. Namely, the mRNA expression level of the key master regulator of adipogenic differentiation nuclear receptor *PPARG* and marker of pre-adipocytes and adipocytes *FABP4* did not change in ADSCs differentiated under the influence of deuterium depleted water (Fig. 4B). Moreover, a statistically significant up-regulation of expression *UCP1* and *ADIPON* genes occurred in the group with low deuterium content compared with the control group and the group with high deuterium content (Fig. 4B). In the group with high deuterium content, significantly lower level of expression of all the studied genes was observed, which indicated inhibition of adipogenic differentiation.

On day 14^th^, the level of *PPARG* and *FABP4* genes expression in the group with high deuterium content reached a level comparable to the groups with natural and low deuterium content. Moreover, the level of gene expression of the key adipokines *LEP* and *ADIPON* was statistically significantly lower than in groups with natural and low deuterium content. Statistically significant increase in the level of *UCP1*, *LPL* and *ADIPON* genes expression and lower level of *FABP4* and *LEP* genes expression compared to the control group on the 14th day was observed. In total, this may reflect the switching the program of adipogenesis in the group with a low deuterium content to the formation of functional thermogenic brown/beige adipocytes.

### Detection of UCP1 expression on protein level by immunocytochemistry

**Figure 5:**
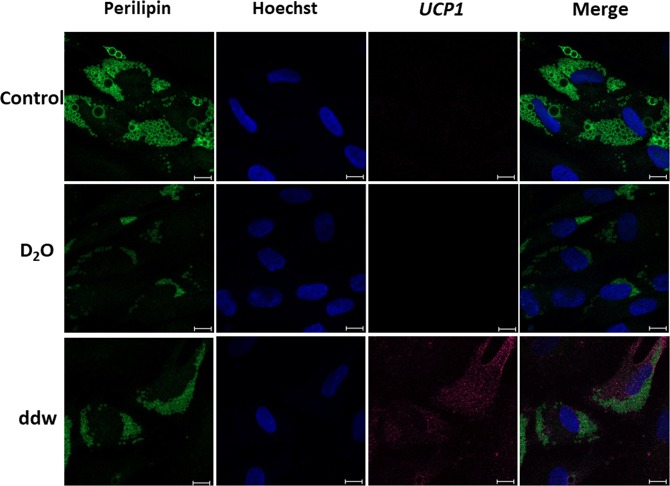
Immunocytochemical detection of *UCP1* in adipocytes, differentiated from ADSCs in media with different deuterium content. Control – differentiation medium was made on the base of milliQ water; ddw – differentiation medium was made on the base of deuterium-depleted water; D_2_O – differentiation medium was made on the base of deuterated water. In each section – the bar = 50 µm. Fluorescent microscopy.

In order to confirm the expression of marker proteins of brown/beige adipocytes at the protein level we performed an immunocytochemical study (Fig. 5).

According to the results of immunocytochemical analysis on the 14th day of ADSCs differentiation using our serum-free adipogenic medium, adipocytes formed and that was confirmed by detection of the adipocyte-specific protein PERELIPIN (forms the membrane of lipid vacuoles). Moreover, only in group with a low deuterium content *UCP1* (a marker of thermogenic brown/beige adipocytes) was detected at the protein level. So, in the group with the natural deuterium content in our serum-free differentiation protocol, the formation of white adipocytes occurred, what was confirmed by the morphological features described earlier (the formation of adipocytes with one or two large lipid vacuoles). In the group with high deuterium content white adipocytes also developed. However, it was noted significant inhibition and delay in time of the adipogenic differentiation process.

### The adipokine production by adipocytes differentiated from ADSCs in media with different deuterium content

Multiplex analysis of 11 key produced adipokines showed that, despite the similar general qualitative spectrum of products, there are statistically significant differences in the quantitative production of cytokines (Table 2). It is important to note that in the group with low deuterium content we observed an increased production of obesity-protective adipokines (LEPTIN and ADIPONECTIN) and IL-6 (this cytokine increases the production of LEPTIN in adipocytes), and decreased production of pro-inflammatory TNF-α, IL-8, MCP-1, IP-10, ADIPSIN (inhibits the lipolysis) and IL-10 (inhibits the thermogenesis and browning). Despite the fact that adipocytes with morphological and phenotypic signs of white adipocytes developed in groups with natural and high deuterium content, statistically significant quantitative differences in the production of all adipokines were also observed between them. It can be explained by significant inhibition and lag of the adipogenesis process in an environment with increased deuterium content.

**Table 2:** The adipokine production by adipocytes differentiated from ADSCs in media with different deuterium content (Control – differentiation medium was made on the base of milliQ water; ddw – differentiation medium was made on the base of deuterium-depleted water; D_2_O – differentiation medium was made on the base of deuterated water).

№	Adipokines,	control, M ± SD (1)	ddw, M ± SD (2)	D_2_0, M ± SD (3)
1	Leptin	57,02 ± 12,77	142,56 ± 23,07	17,26 ± 8,53
			p_1–2_ = 0,0021	p_1–3_ = 0,0003
				p_2–3_ = 0,0001
2	Adiponectin	67,19 ± 15,53	175,12 ± 30,90	33,17 ± 13,77
			p_1–2_ = 0,0048	p_1–3_ = 0,0261
				p_2–3_ = 0,0008
3	Adipsin	63,66 ± 16,20	35,36 ± 3,76	91,52 ± 16,24
			p_1–2_ = 0,0228	p_1–3_ = 0,0659
				p_2–3_ = 0,0011
4	Vaspin	39,29 ± 14,07	16,83 ± 2,86	0,92 ± 0,38
			p_1–2_ = 0,0293	p_1–3_ = 0,0034
				p_2–3_ = 0,0004
5	Chemerin	77,82 ± 11,80	20,12 ± 3,59	16,99 ± 7,28
			p_1–2_ = 0,0005	p_1–3_ = 0,0003
				p_2–3_ = 0,5052
6	TNF-α	17,89 ± 0,16	12,27 ± 0,77	0,87 ± 0,39
			p_1–2_ = 0,0001	p_1–3_ = 0,0001
				p_2–3_ = 0,0001
7	IL-6	28,14 ± 5,97	106,60 ± 40,41	11,66 ± 1,98
			p_1–2_ = 0,0138	p_1–3_ = 0,0068
				p_2–3_ = 0,0008
8	IL-8	46,26 ± 10,89	21,29 ± 6,34	73,49 ± 7,97
			p_1–2_ = 0,0065	p_1–3_ = 0,0093
				p_2–3_ = 0,0002
9	IL-10	11,15 ± 1,69	5,63 ± 1,78	25,59 ± 8,41
			p_1–2_ = 0,0191	p_1–3_ = 0,0135
				p_2–3_ = 0,0059
10	MCP-1	30,68 ± 6,77	1,76 ± 0,92	5,51 ± 2,31
			p_1–2_ = 0,0024	p_1–3_ = 0,0008
				p_2–3_ = 0,0203
11	IP-10	8,12 ± 2,61	1,78 ± 1,51	3,24 ± 2,16
			p_1–2_ = 0,0446	p_1–3_ = 0,0348
				p_2–3_ = 0,7377

### Viability and metabolic activity of ADSCs in the process of adipogenesis in differentiation media with various deuterium content

To clarify the question whether deuterium-mediated inhibition of ADSCs adipogenic differentiation was associated with cytotoxic effect of deuterium we analyzed viability and metabolic activity of differentiated ADSCs.

### Viability of ADSCs in inductive media with various deuterium content

The cell viability/cytotoxicity was assessed with FDA and PI staining ADSCs before differentiation and during differentiation in adipogenic media with various deuterium content on 3, 7 and 14 days of cultivation (Table 3). The cell viability in culture of ADSCs in growth medium before differentiation was 97.3 ± 3.4% according to FDA/PI staining. On the 3rd day of differentiation, a statistically significant decrease in viability was observed in all groups, which can be caused by apoptosis and necrosis of part of the cells upon transition to both serum-free culturing conditions and apoptosis under the influence of differentiation factors. It is important to note that in the group with a high deuterium content on the 3rd day of the adipogenesis, there was a sharp decrease in cell viability, which was probably due to the cytotoxic effect of deuterium in the differentiating microenvironment. On the 7th and 14th days in a group with a high deuterium content, gradual increase in cell viability occurred, probably due to the adaptation of cells to a high deuterium content in the microenvironment.

**Table 3:** The viability values of ADSCs cultured in media with various deuterium content after 7 and 14 days of cultivation (Control – differentiation medium was made on the base of milliQ water; ddw – differentiation medium was made on the base of deuterium-depleted water; D_2_O – differentiation medium was made on the base of deuterated water) (FDA/PI assay).

Exposition	Viability, % (by FDA/PI assay)		
	Control	ddw	D_2_O
3 day	93.5 ± 1.9	93.4 ± 2.3	83.8 ± 5.2^*,#^
7 day	94.5 ± 2.4	95.3 ± 3.2	87.7 ± 6.3^*,#^
14 day	93.7 ± 1.8	92.1 ± 2.6	91.3 ± 2.3

^*^The results are expressed as mean ± SD (n = 6), significant differences between the experimental group *p < 0.05 compared to control group, #p < 0.05 compared to ddw group.

### Metabolic activity of ADSCs in inductive media with various deuterium content

**Figure 6:**
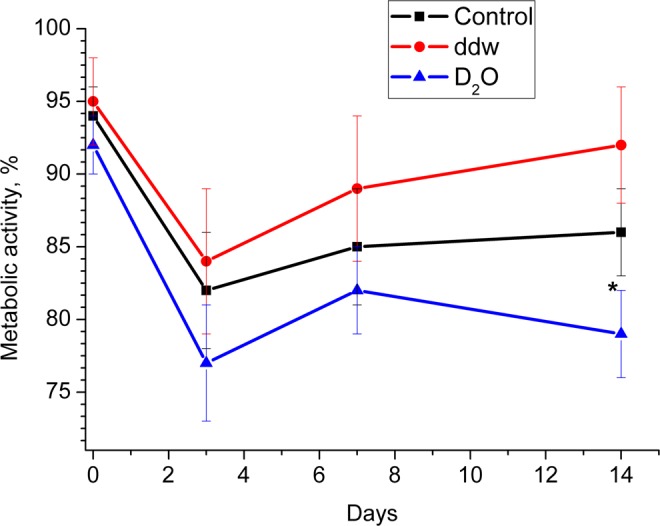
Diagrams represent results of the Alamar Blue assay (Metabolic activity) in media with various deuterium content after 0, 3, 7 and 14 days. Control – differentiation medium was made on the base of milliQ water; ddw – differentiation medium was made on the base of deuterium-depleted water; D_2_O – differentiation medium was made on the base of deuterated water. (The results are expressed as mean ± SD (n = 6), significant differences between groups: *p < 0.05 compared to control group).

Alamar Blue assay was performed on 3^rd^, 7^th^ and 14^th^ day of ADSCs differentiation in adipogenic media with various deuterium content (Fig. 6). The metabolic activity of undifferentiated ADSCs cultured in growth medium was set at 100%, and their changes are given as percentage of controls.

A decrease in metabolic activity in all groups on the 3rd day of adipogenesis was observed, what may be both due to switching the cells from growth (proliferation) processes to differentiation, and to adaptation to serum-free conditions. Subsequently, in groups with a natural and low deuterium content, an increase in metabolic activity occurred, especially pronounced in the group with low deuterium content. This fact may reflect the differentiation of ADSCs in brown/beige adipocytes when using our cocktail of differentiating factors in a microenvironment with low deuterium content. Importantly, metabolic activity was reduced in the group with a high deuterium content compared to the group with a natural and low deuterium content during the entire adipogenesis. It correlates with morphological characteristics and gene expression data and probably can be explained by the cytotoxic effect of deuterium at the induction stage/committing cells and its inhibitory effect on the differentiation of ADSCs.

## Discussion

Obesity, metabolic syndrome and T2DM became a global public health problem in the 21st century. One of the key links in the pathogenesis of the development of these diseases is adipose tissue. In the last 10 years, the phenomenon of the existence of active thermogenic beige adipose tissue in adult humans was discovered. The possibility of *in vivo* conversion of subcutaneous white adipocytes into beige adipocytes was further shown. These facts, in total, in addition to upcoming pharmacological strategies for inhibiting the development of adipose tissue, can also be considered as a promising approach for the prevention and treatment of obesity, metabolic syndrome and T2DM by the formation of beige adipose tissue *de novo* and/or by the conversion of existing subcutaneous white adipocytes into beige adipocytes.

In our study, the directed adipogenic differentiation of ADSCs with a two-step (induction + maturation) non-serotonous protocol based on the use of various hormones and small molecules led to the formation of classical white adipocytes in a microenvironment with a natural and high deuterium content. Interestingly, adipogenesis in a low deuterium content is diverted towards the development of brown/beige adipocytes under the influence of the same induction and differentiation factors. The differentiation of ADSCs in brown/beige adipocytes in microenvironment with low deuterium content was confirmed by morphological features, gene expression, *UCP1* protein detection. Moreover, despite the fact that the resulting brown/beige adipocytes produced the same spectrum of adipokines as their counterparts (white adipocyte) obtained in the process of differentiation of ADSCs in microenvironment with natural and high deuterium content, but there were significant quantitative differences. In general, brown/beige adipocytes showed increased production of anti-obesity and lower production of pro-inflammatory adipokines. Thus, in addition to forming of beige adipocytes, which are functionally useful for the prevention and treatment of obesity, metabolic syndrome and T2DM, we also discovered the secretion of a protective spectrum of adipokines, which can provide additional benefits at the systemic level of the whole body (organism).

Despite the difference of morphology and resulting type of differentiated from ADSCs adipocytes (brown/beige *vs* white), both ddw and D_2_O groups inhibited adipogenic differentiation as confirmed by gene expression and Red Oil O extraction assay. However, the inhibition of adipogenesis in D_2_O group was drastic which can be partially explained by acute deuterium cytotoxicity. In our previous study on the effect of deuterated and deuterium-depleted water on the proliferation and migration of ADSCs, the acute cytotoxic effect of high deuterium concentrations was also noted^41^. An interesting question for further research is to establish the mechanism of cell death (necrosis vs apoptosis) with high deuterium content, as well as the role of mitochondria in cell death. A comparative study of the mechanisms of cell death in conditions that promote growth (proliferation) and differentiation is also interesting. In case of ddw group, cell viability was not affected. This is in line with our previous observations as well as with other authors’ reports^41,52–55^.

At the same time, deuterium excess or depletion had opposite effect on the cellular metabolism: differentiated ADSCs metabolic activity was increased in ddw group and inhibited in D_2_O group (Fig. 6). This also corresponds to our earlier data on the effect of low and high deuterium content on the metabolic activity of ADSCs in growth conditions^41^.

High concentrations of deuterium in the environment can lead to inhibition of many reactions associated with the energy component in the chain of biochemical reactions^20,22,23^. This can be explained by the fact that hydrogen is the main participant in electron transfer and is a reducing agent in many biochemical cascades^30,35,37^. Therefore, stronger deuterium bonds, when replacing protium, will lead to inhibition of such reactions.

As for deuterium depletion, after classical ideas about the dilution of substances^56^, changes in the metabolic rate should be negligible at deuterium concentration below 150 ppm. However, the observed effects are significant^57^. This suggests that deuterium acts as an element, which is necessary as a rate regulator of biochemical reactions cascades. On the other hand, deuterium could be considered as an element that affects the substance chirality. That explains the mechanism of changes in many system parameters as a result of different D/H ratios^22,27,41^. In other words, the presence of deuterium or protium in a substance leads to various ways of further reactions. Accordingly, the entire metabolism can go in different (and unpredictable yet) directions (at different rates) depending on the presence of deuterium or protium in the initial or intermediate substance.

In order to find out some mechanisms of the action of deuterium on the ADSCs adipogenic differentiation, we examined the mRNA expression of some adipogenesis markers.

So, peroxisome proliferator-activated receptor gamma (PPAR-γ or *PPARG*) is a master gene (master regulator) of adipogenic differentiation and encodes a type II nuclear receptor. *PPARG* is mainly presented in adipose tissue, colon and macrophages and regulates fatty acid storage and glucose metabolism^58–60^. Here we did not observe significant changes in the *PPARG* expression in all comparison groups. But we observed inhibition of express *PPARG* gene at the stage of induction of adipogenesis in groups with both high and low deuterium content. Moreover, at the end of the differentiation process, the level of expression increase, which may be explained by the asynchrony of the differentiation process in the ADSCs population at the single cells level.

*FABP4* (fatty acid binding protein 4) or aP2 (adipocyte Protein 2) is a carrier protein for fatty acids that is primarily expressed in adipocytes and macrophages^61^. Blocking this protein is perspective for treating various diseases^62–66^. After our data, *FABP4* expression was slightly reduced in both groups at the same time the level of *PPARG* gene expression reflected the overall delay in the process of ADSCs differentiation.

Leptin (*LEP*) is a hormone predominantly made by adipose cells that helps to regulate energy balance by inhibiting hunger. Leptin expression level in both groups was slightly lower than in the control group. This suggests that the level of cell metabolism, namely, the absorption of lipids, takes place at low intensity. A high concentration of *LEP* in the medium with reduced expression of the leptin gene compared with the control group can be explained by different rates of leptin secretion, which, as is known from work^67^, is independent of the regulation of leptin mRNA expression due to the presence of vesicular leptin depots in adipocytes^68^. Moreover, since adipocytes themselves express leptin receptors^69^, hypothetically, this difference can also be associated with differences in autocrine signaling between different adipocyte subtypes.

Adiponectin (*ADIPON*) is a hormone that regulates glucose metabolism and fatty acid oxidation^70^. Some studies have shown that *ADIPON* was inversely correlated with body weight index^71^. Studies of mice with elevated levels of adiponectin showed a decrease in adipocyte differentiation and increase in energy costs, which was due to the uncoupling of mitochondria^72^. It has also been shown that adiponectin and leptin alter insulin sensitivity in mice^73^. High *ADIPON* expression in ddw group could be associated with high metabolism level and high mitochondrial activity in context of ADSCs differentiation in this condition into beige adipocytes^35,37,41^. Another cause of high adiponectin expression could be a compensatory effect to low leptin expression as these two proteins act synergistically^73,74^. The low *ADIPON* expression in the D_2_O group may be associated with both general inhibition of the adipogenesis process and deviation of the direction of differentiation into white adipocytes with switching to energy storage by phosphorylation of fatty acids^75^.

Lipoprotein lipase (*LPL*) is a water-soluble enzyme that hydrolyzes triglycerides in lipoproteins. It is also involved in promoting the cellular uptake of chylomicron remnants, cholesterol-rich lipoproteins, and free fatty acids^76–78^. In the deuterated medium, we did not observe *LPL* expression on day 7^th^ of the experiment, and, it was significantly lower on day 14^th^. This may be explained by deuterium-triggered metabolic changes which led to the formation of white adipocytes. In the ddw medium there was no difference with the control group.

*UCP1* (uncoupling protein 1, also known as thermogenin)^79–81^ is an uncoupling protein found in the mitochondria of beige adipose tissue (BAT). Heat production using *UCP1* in beige adipose tissue occurs with the separation of cellular respiration and phosphorylation, that is the rapid oxidation of nutrients occurs with a low intensity of ATP production^82^. According to our data, the decreased expression of *UCP1* in a deuterated medium confirms that differentiation of ADSCs in this condition goes towards white adipocytes. High expression of *UCP1* in ddw medium seems to be the result of the ADSCs differentiation into functional beige adipocytes. Consequently, the main mechanism of the inhibitory/stimulating effect of high/low deuterium concentrations should be sought in the work of the respiratory chain and the mitochondrial complex itself.

Taking into account the fact that ddw directs the differentiation of adipocytes into their other subtype (brown/beige vs white adipocytes), the apparent “inhibition” of adipogenesis in the ddw group compared to the control medium actually reflects the differences in the formation of different adipocyte subtypes. Whereas in the case of a group with a high deuterium content compared with the control, the inhibition process of differentiation of white adipocytes from ADSCs actually occurs.

In our study, we first obtained data on the effect of various deuterium concentrations on the efficiency and direction (brown/beige vs white adipocytes) of adipogenic ADSCs differentiation in an *in vitro* model system. For the possible practical application of these results, additional studies are needed that would allow a more detailed description of the molecular mechanisms of influence of deuterium various concentrations at the cellular level, as well as studies at the body level. In further experiments on laboratory animals, it is necessary to study the effect of low concentrations of deuterium on the functioning of various fat depots in various physiological and pathological situations (stress, high-fat diet, etc.)^83,84^.

Altogether, our data revealed the importance of D/H ratio in culture medium for directed adipogenic differentiation of human ADSCs. However, the mechanisms that explain involvement different deuterium concentration in regulation ADSCs adipogenic differentiation are not clarified and this opens a field for the future research.

## Conclusion

We have demonstrated that both deuterium depleted and deuterated inductive media inhibit adipogenic differentiation of human ADSCs compared to medium with normal deuterium content. The inhibitory effect of deuterated medium could be partially explained by its increased cytotoxicity. However, surprisingly, with the same instructional signals (hormones and small molecules), the differentiation of ADSCs in the microenvironment with a low deuterium content deviated in direction of brown/beige adipocytes development. Thus, the separate or combined use of ddw and D_2_O may be used in the complex treatment of obesity, metabolic syndrome and T2DM.

## Data Availability

The data used to support the findings of this study is available from the corresponding author upon request.
